# Exposure to e-Cigarette Posts Across Social Media Platforms and Its Associations With Susceptibility and e-Cigarette Use: Comparative Cross-Sectional Study of High Schoolers in Jalisco (Mexico) and Southern California (United States)

**DOI:** 10.2196/85376

**Published:** 2026-03-13

**Authors:** Dèsirée Vidaña-Perez, Inti Barrientos-Gutierrez, Rosibel Rodríguez-Bolaños, Evangelina Díaz-Andrade, Diego F Leal, Minji Kim, Jennifer B Unger, Thomas W Valente, Jessica L Barrington-Trimis, James F Thrasher

**Affiliations:** 1 Department of Health Promotion, Education, and Behavior Arnold School of Public Health University of South Carolina Columbia, SC United States; 2 Evaluation and Surveys Research Center National Institute of Public Health Cuernavaca, Morelos Mexico; 3 Department of Reproductive Health Population Health Research Center National Institute of Public Health Cuernavaca, Morelos Mexico; 4 Health Region VI, Ciudad Guzman, Jalisco Health Services Cd. Guzman Mexico; 5 University Center of the South Universidad de Guadalajara Guadalajara Mexico; 6 School of Sociology University of Arizona Tucson, AZ United States; 7 Department of Population and Public Health Sciences University of Southern California Los Angeles, CA United States

**Keywords:** social media use, e-cigarette use, e-cigarette susceptibility survey research, adolescents, vaping

## Abstract

**Background:**

Adolescents’ exposure to electronic cigarette (e-cigarette) content through social media platforms influences their perceptions and behaviors, although cross-country analyses in different regulatory environments are scarce.

**Objective:**

This study evaluated the association between e-cigarette exposure on social media platforms and e-cigarette susceptibility and use in Jalisco (Mexico) and Southern California (United States).

**Methods:**

In 2022-2023, students from 23 high schools in Jalisco (n=1418) and 11 in Southern California (n=2953) were surveyed with harmonized measures on past-month frequency of social media platform use (ie, YouTube [Google], Instagram [Meta], TikTok [ByteDance], Snapchat [Snap Inc], WhatsApp [Meta], Facebook [Meta], Twitter (now “X”), and Twitch [Twitch Interactive]) and seeing e-cigarette posts on each social media platform used; which were recoded to 5-point scores (range 0-4) for social media platforms use and e-cigarette post exposure. Country-stratified logistic models regressed e-cigarette susceptibility (among noncurrent users) and past-month use on social media platform scores, adjusting for age, sex, family affluence, and friends’ e-cigarette use.

**Results:**

Past-month e-cigarette use was higher in Jalisco (248/1418, 17.5%) than Southern California (139/2953, 4.7%; *P*<.001). Social media use and e-cigarette exposure on each social media platform differed across samples (*P* values<.001). In Southern California, more frequent social media use was positively associated with e-cigarette susceptibility (adjusted odds ratio [AOR] 1.83, 95% CI 1.48-2.25), whereas in Jalisco, higher frequency of exposure to e-cigarette content was associated with susceptibility (AOR 1.21, 95% CI 1.02-1.43). Higher frequency of social media use and exposure to e-cigarette content were both positively associated with past-month e-cigarette use in Southern California; in Jalisco, greater exposure to social media platforms and e-cigarette content was associated with past-month use.

**Conclusions:**

Frequent social media platform use and e-cigarette exposure through social media platforms appear to be associated with e-cigarette susceptibility and use across contexts. Stronger policies to limit and enforce online exposures are needed.

## Introduction

Over the last decade, adolescent cigarette use has significantly decreased in many countries around the world that have adopted strong tobacco control policies, including bans on cigarette advertising through traditional media channels [1,2]. In contrast, adolescents’ use of electronic cigarettes (e-cigarettes) has increased as countries have been slower to adopt and enforce strong e-cigarette regulations. Moreover, increases in e-cigarette use have coincided with and appear to be partly explained by the growing and widespread use of social media platforms, which is often the primary means of adolescent exposure to e-cigarette marketing [3].

e-Cigarettes were first marketed as a harm reduction product to help adult smokers quit [4], yet they have become increasingly popular among adolescents in both high and middle-income countries with different regulatory frameworks. In the United States, for example, adolescent e-cigarette use rapidly increased in 2017-2018, which prompted policy actions, including federal prohibition of e-cigarette sales to anyone younger than 21 years in December 2019 [5]. Furthermore, in 2020, the US Food and Drug Administration (FDA) banned cartridge-based e-cigarettes with any flavors except tobacco or menthol, and the FDA has also denied marketing requests for most e-cigarette products [6]. Despite enforcement issues and loopholes for some e-cigarette types (eg, disposables), these measures have been accompanied by decreased current e-cigarette use among high schoolers from 20.8% in 2018 to 10% in 2023 [7,8]. However, e-cigarettes remain the most commonly used tobacco product among US adolescents [7]. These trends generally characterize adolescents in California (United States), although the prevalence of e-cigarette use is lower there than in other states [9].

In Mexico, the 2008 General Tobacco Control Law stipulated that no new tobacco products or any products that resemble tobacco products could be introduced or sold in the country [10]. Regulators interpreted this legislation as a de facto ban on e-cigarette marketing and sales, with a 2020 Presidential Decree specifically banning their importation and clarifying that the General Tobacco Control Law banned e-cigarette marketing and sales [11]. However, the national prevalence of past-month e-cigarette use among 15- to 18-year-olds increased from 2% in 2021 to 3.3% in 2022 [12]. Even though the prevalence is relatively low compared with the United States, the increase from 2021 to 2022 represents a 65% growth in use. Furthermore, representative data from urban public schools in large Mexican cities suggest that e-cigarette use is substantially higher than these estimates [13], perhaps due to reduced bias from adolescents’ self-administering surveys in schools compared with the national surveys that involve survey administration in households. Importantly, despite Mexico’s e-cigarette ban, e-cigarettes are accessible through a variety of venues in both the formal and informal economy due to a lack of enforcement [14,15]. Illegal marketing through social media platforms—which is particularly challenging to enforce—may also contribute to Mexican adolescents’ e-cigarette use.

The growth in internet access has been accompanied by increasing use of social media platforms worldwide [16]. In Mexico, 96% of adolescents and young adults reported using social media platforms in 2021 [17], and in 2022, 97% of US adolescents aged between 13 and 17 years reported using them [18]. Furthermore, in 2023, it was estimated that 77% of US high-school students used social media platforms several times a day [19]. Among youth in Canada, England, and the United States, those in the United States reported the greatest increase in noticing e-cigarette marketing from 2017 to 2019, with “websites or social media” being the second most reported channel of marketing exposure [20]. Additionally, a 2021 survey among middle- and high-school students in the United States found that 73.5% of social media users reported having ever seen e-cigarette–related content on social media platforms [21]. To our knowledge, there are no studies in Mexico or other Latin American countries where e-cigarettes are banned that have assessed exposure to e-cigarette content across social media platforms and its associations with susceptibility or use of these products.

e-Cigarette advertisements on social media platforms usually portray the devices and their use as desirable, trendy*,* and fashionable, which likely appeals to adolescents [22]. Indeed, exposure to e-cigarette advertisements has been associated with more positive perceptions of vaping, lower harm perception, change in perceived acceptability among peers [23], as well as with greater susceptibility to and current use of e-cigarettes among both adolescents and young adults [24,25]. To maximize attention and engagement, many e-cigarette brands use social media *influencers* or celebrities to promote their products [22]. Even though advertisements on social media platforms can be targeted to specific population groups (ie, >18 y), 1 study found that more than 60% of the e-cigarette brands and *influencers* on Instagram [Meta] had no age-gating restrictions for followers, although their posts were intended only for adult users [26]. A key feature of social media concerns its transnational reach across jurisdictional borders, making it possible for adolescents in one country to access content from other countries, even if the content contains marketing that is illegal in their home countries.

This study examined similarities and differences in the use of social media platforms, exposure to e-cigarette posts on social media platforms, and their associations with e-cigarette susceptibility and use among high schoolers from the southern part of Jalisco in Mexico and Southern California in the United States. As described above, tobacco regulations and adolescent e-cigarette use prevalence differ between Mexico and the United States, with potential consequences for adolescents’ exposure to e-cigarette content via social media platforms. Furthermore, a higher proportion of the Mexican American population live in Southern California [27], which may help reduce differences in the study populations due to cultural background. We expected lower exposure and use among Mexican adolescents due to the national e-cigarette ban. Nevertheless, in both countries, we expected that greater exposure to e-cigarette posts on social media platforms would be positively associated with susceptibility and use, even after adjusting for overall social media platform use. To our knowledge, this is the first study of adolescent exposure to e-cigarette content across specific social media platforms and the association with susceptibility and use across countries.

## Methods

### Population

Data for Mexico come from the 2022-2023 Mental Health, Addiction, and Violence Survey in the state of Jalisco. A convenience sample of high schools (n=23) in 16 municipalities of the Southern part of Jalisco answered an online, self-administered survey from November 2022 to February 2023. A total of 1557 students were invited to participate, 131 (8.4% approximately) refused, yielding a sample of n=1426. US data come from the Assessing Developmental Patterns of Vaping, Alcohol, Nicotine, and Cannabis Use and Emotional Wellbeing (ADVANCE) cohort [27] in Southern California; participants were recruited as ninth-grade students in either 2020 (class of 2024) or 2021 (class of 2025) from 11 high schools. To harmonize survey timing with Mexico, we analyzed data only from the February to June 2023 survey, when the Southern California students were in 10th or 11th grade. A total of 4206 students were invited to participate, 631 (15% approximately) refused, yielding a sample of 3575. Participants with missing data were dropped from analyses (Jalisco final sample, n=1418, vs Southern California final sample, n=2953).

### Ethical Considerations

Before survey administration, both studies obtained students’ assent, and ADVANCE also obtained parental consent. For the Mental Health, Addiction, and Violence Survey in the state of Jalisco, active parental consent was not required from the ethics committee; parents were informed about the study and could request that their child be excluded. Protocols were approved by the Institutional Review Board and Ethics Committee of the Ministry of Health of Ciudad Guzmán, Jalisco, Mexico (103/RVI/2021), and for Southern California, by the Institutional Review Board of University of Southern California (HS-19-00682). Participants in ADVANCE received a small token of appreciation (eg, stickers or a pen). Absentee and transfer students were offered a US $10 gift card to complete the survey independently. No incentives were provided for participants in Jalisco. Projects are hosted on secure servers. To protect participants’ privacy, data were deidentified before sharing. When data transfer was required, a 2-step secure procedure was used. First, a password-protected link to the REDCap (Research Electronic Data Capture; Vanderbilt University) server was sent to the authorized recipient. The password was then transmitted separately in a second email. The download link was single-use and remained active for a limited time to further enhance security.

### Measurements

#### Social Media Platforms–Related Variables

To assess general social media engagement, students were asked, “How often do you visit the following social media sites?” for each of the following social media platforms—Facebook [Meta], Instagram, WhatsApp [Meta; Jalisco only], Snapchat [Snap Inc; Southern California only], Twitter (now “X” [X Corp] as of July 24, 2023), YouTube [Google], TikTok [ByteDance], and Twitch [Twitch Interactive]. Response options in both surveys were (1) 0=“I don’t use this social media,” (2) 1=“once a month or less,” (3) 2=“weekly,” (4) 3=“daily,” (5) 4=“several times a day,” and (6) 5=“I don’t know.”

Next, to assess exposure to e-cigarette content on social media, students were asked, “How often do you see posts about e-cigarettes and nicotine vaping products on the following social media sites (same as above)?” In Jalisco responses were “I don’t use this social media,” “never,” “once a month or less,” “weekly,” “daily,” and “several times a day” while in Southern California responses were “never,” “monthly or less,” “weekly,” “daily,” “several times a day,” and “ I don’t know”. To harmonize responses across samples, first we recoded “I don’t use this social media” (Jalisco) and “don’t know” (Southern California) along with “never” as 0, indicating no exposure; then we rescaled all remaining responses to a 0-4 scale for comparability (0=“I don’t use this social media, never, or I don’t know,” 1=“once a month or less,” 2=“weekly,” 3=“once a day,” and 4=“several times a day”). Finally, we calculated 2 continuous scores, that is, social media use score and e-cigarette postexposure score by averaging responses across platforms (range=0-4) to reflect students’ average frequency of social media use and exposure to e-cigarette–related content, respectively.

#### e-Cigarette Use and Susceptibility

Students reported their frequency of using e-cigarettes in the previous 30 days (“In the last 30 days, how many total days have you used any electronic cigarette with nicotine?”). Responses were recoded into a dichotomous variable (0=“No use in the past 30 days” [noncurrent users], and 1=“any use in the past 30 days”). Susceptibility to use e-cigarettes was assessed with the question “Do you think that at some point during the next 12 months, you will use an e-cigarette?” with responses recoded as a dichotomous variable based on previous research on susceptibility among youth [28] (0=“definitely not, 1=“probably not,” “probably yes,” or “definitely yes”).

### Covariates

Sociodemographic variables included age (12 y, 13 y, 14 y, 15 y, 16 y, 17 y, 18 y, 19 y or older), sex (female and male), and household wealth, using the Family Affluence Scale (FAS). The FAS includes the following questions, (1) how many cars or trucks does your family own (0, 1, 2 or more); (2) do you have a room for yourself? (0 or 1); (3) during the last 12 months, how many times did you go on vacation with your family? (0, 1, 2, 3 or more); (4) how many computers does your family have? (0, 1, 2, 3 or more). The FAS has shown reliability and validity as a measure of socioeconomic status [29], in both the United States [30] and Mexico [31]. As is standard practice, the FAS items were summed (range=0-9), with higher scores indicating higher family affluence. Use of e-cigarettes by friends was also assessed (Jalisco: “Of your five best friends, how many use electronic cigarettes?” and Southern California: “How many of your five (5) closest friends use electronic cigarettes for vaping nicotine?”) with responses dichotomized to indicate having any friends who use (0=none, and 1=1-5).

### Analyses

For descriptive analyses, datasets were combined to allow comparison of sample characteristics using Pearson chi-square tests for categorical variables and independent sample *t* tests for the continuous variables. We used Pearson chi-square tests to assess if there were any differences in the frequency of social media platform use and frequency of exposure to e-cigarette content through social media platforms across countries. Then, for each country, we conducted crude and adjusted logistic regression models with Standard Errors (SEs) clustered by school to account for intragroup correlation among students within schools were estimated for each of two outcomes, (1) susceptibility to use e-cigarettes (analytic sample=noncurrent users [n=1170 for Jalisco, and n=2695 for Southern California]) and (2) current e-cigarette use (analytic sample=entire sample [n=1418 for Jalisco, and n=2953 for Southern California]). Independent variables included the index of frequency of exposure to e-cigarette posts on social media, as well as the frequency of social media use index and other covariates. Due to potential different effects per social media platform [28], sensitivity analyses were conducted by analyzing one social media platform at a time, adjusted by the frequency of social media use (Multimedia Appendix 1). To further assess the robustness of findings to alternative methods for handling within-school correlation, additional sensitivity analyses were conducted using multilevel logistic regression models with school specified as a random effect. Results from multilevel models were consistent in direction, magnitude, and statistical significance with those from the clustered SE approach; therefore, clustered models were retained as the primary analyses (Multimedia Appendix 2). Finally, pooled analyses across countries were conducted using adjusted models for susceptibility and use that included an interaction between country and exposure to e-cigarette content via social media. These pooled models were treated as supplementary to country-stratified analyses due to differences in the measurement of key variables across countries (Multimedia Appendix 3). All analyses were conducted using Stata (v.19; StataCorp LLC).

## Results

Compared with the Southern California sample, the Jalisco sample was younger, more likely to be male, and had lower family affluence (Table 1). Susceptibility and past-month e-cigarette use were higher in Jalisco (vs Southern California), as were the number of friends using e-cigarettes, frequency of social media exposure, and frequency of e-cigarette exposure on social media platforms.

**Table 1:** Sample characteristics of high-school students in Jalisco (Mexico) and Southern California (United States).

Characteristics			Jalisco (N=1418^a^)		Southern California (N=2953^a^)		*P* value
Age (y), mean (SD)			15.9 (0.97)		16.7 (0.58)		<.001
**Sex, n (%)**							<.001
	Female	887 (62.5)		1656 (56.1)			
	Male	531 (37.5)		1297 (43.9)			
Family Affluence Scale (0-9), mean (SD)			4.10 (2.10)		5.60 (1.81)		<.001
**e-Cigarettte use, n (%)**							<.001
	Not current, not susceptible	830 (58.5)		2469 (83.6)			
	Not current, susceptible	340 (24)		340 (11.5)			
	Past-month use	248 (17.5)		139 (4.7)			
Best friends’ use of e-cigarettes, n (%)			785 (55.4)		730 (24.7)		<.001
Frequency of using social media (range=0-4), mean (SD)			2.1 (0.77)		1.6 (0.66)		<.001
Frequency of e-cigarette exposure on social media (range=0-4), mean (SD)			0.66 (0.83)		0.36 (0.56)		<.001

**^a^**Sample for Jalisco included 23 high schools; Southern California included 11 high schools.

**Figure 1:**
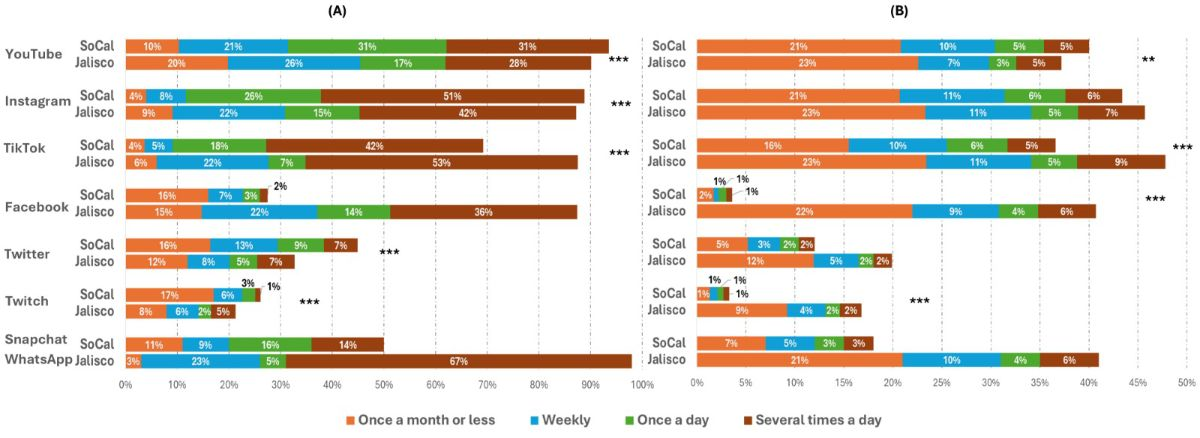
(A) Frequency of using social media, high school students in Jalisco and Southern California, and (B) frequency of e-cigarette exposure on social media, high school students in Jalisco and Southern California. Snapchat and WhatsApp were not compared since they were not included in both samples. Significance of *P* values: **.05, ***<.001. SoCal: Southern California.

WhatsApp was the most frequently used platform among those assessed in Jalisco, with 67% (n=950) of students reporting its use several times a day (not asked in Southern California). In Jalisco, among the social media platforms queried in both countries (Figure 1A), the frequency of use reported was significantly different between countries for YouTube, Instagram, TikTok, Facebook, Twitter, and Twitch (*P*<.001 for all). YouTube was the most frequently used in both sites; however, most Southern California students reported using it once a day (907/2953, 31%) or several times a day (918/2953, 31%) while fewer Jalisco students reported the same (235/1418, 17% once per day; 399/1418, 28% several times per day). Facebook was frequently used in Jalisco (710/1418, 50% at least once a day), while fewer than 30% (799/2953) of students reported any use of that platform in Southern California. Twitch was the least used platform in both samples.

The frequency of e-cigarette exposure on each social media platform differed across samples, except for Instagram (Figure 1B). TikTok was the platform through which Jalisco students reported the greatest exposure, with 25% (333/1418) reporting seeing e-cigarette content at least weekly (153/1418, 11% weekly; 66/1418, 5% once per day; and 127/1418, 9% several times per day). Instagram had the highest self-reported exposure to e-cigarette posts in Southern California, with at least 21% (620/2953) reporting weekly exposure (313/2953, 11% weekly; 185/2953, 6% once per day; and 169/2953, 6% several times per day). The lowest frequency of e-cigarette exposure for Southern California was through Twitch and Facebook, and exposure was lowest through Twitch and Twitter in Jalisco.

For each sample, separate bivariate and multivariate adjusted logistic regression models were estimated for e-cigarette susceptibility among high schoolers who did not use e-cigarettes in the last month (Table 2). In unadjusted and adjusted models for both Jalisco and Southern California, females had lower odds of being susceptible than males (adjusted odds ratio [AOR] 0.62, 95% CI 0.47-0.83; AOR 0.60, 95% CI 0.52-0.71, respectively), and those whose best friends used e-cigarettes had higher odds of susceptibility (AOR 3.80, 95% CI 2.87-5.03; AOR 3.35, 95% CI 2.48-4.52, respectively). In bivariate models for both samples, higher frequency of social media use and of exposure to e-cigarette content via social media were positively associated with susceptibility; however, in adjusted models, more frequent social media use was positively associated with susceptibility in Southern California (AOR 1.83, 95% CI 1.48-2.25) but not in Jalisco. In adjusted models, greater frequency of exposure to e-cigarette content on social media was independently associated with susceptibility only in Jalisco (AOR 1.21, 95% CI 1.02-1.43).

**Table 2:** Unadjusted and adjusted logistic models for susceptibility to use e-cigarettes (among those with no e-cigarette use in the past 30 days), high school students in Jalisco and Southern California, 2022-2023.

Variables			Jalisco, Mexico (n=1170)									Southern California, United States (n=2695)						
			OR^a^ (95% CI)		*P* value		AOR^b^ (95% CI)		*P* value		OR (95% CI)			*P* value		AOR (95% CI)		*P* value
Frequency of using social media, index^c^			1.28 (1.08-1.51)		<.001		1.13 (0.94-1.36)		.16		2.08 (1.72-2.51)			<.001		1.83 (1.48-2.25)		<.001
Frequency of e-cigarette exposure on social media, index^d^			1.31 (1.12-1.52)		.002		1.21 (1.02-1.43)		.03		1.52 (1.33-1.75)			<.001		1.15 (0.98-1.32)		.18
Age			1.06 (0.93-1.20)		.41		1.06 (0.92-1.21)		.45		1.08 (0.82-1.42)			.45		1.02 (0.79-1.31)		.85
**Sex**																		
	Male	*Ref* ^e^				*Ref*				*Ref*					*Ref*			
	Female	0.55 (0.43-0.74)		<.001		0.62 (0.47-0.83)		<.001		0.52 (0.46-0.58)			<.001		0.60 (0.52-0.71)		<.001	
Family affluence scale (0-9)			0.95 (0.90-1.01)		.09		0.98 (0.92-1.05)		.64		1.06 (0.98-1.15)			.07		1.06 (0.98-1.15)		.10
**Best friends’ use of e-cigarettes**																		
	None	*Ref*				*Ref*				*Ref*					*Ref*			
	1 or more	4.09 (3.11-5.39)		<.001		3.80 (2.87-5.03)		<.001		3.98 (2.94-5.37)			<.001		3.35 (2.48-4.52)		<.001	

^a^OR: odds ratio.

^b^AOR: adjusted odds ratio from model that includes all variables shown in table.

^c^Frequency of using social media index represents the average of the reported frequency of using each social media platform, it ranges from (0-4).

^d^Frequency of seeing e-cigarette posts on social media index represents the average of the reported frequency of seeing e-cigarettes posts in each social media platform, ranges (0-4).

^e^*Ref*: reference value.

In unadjusted and adjusted models for past-month e-cigarette use in both samples (Table 3), a higher frequency of exposure to e-cigarette content on social media (Jalisco: AOR 1.49, 95% CI 1.27-1.74; and Southern California: AOR 1.51, 95% CI 1.15-1.73) was positively associated. Elevated odds were also observed for higher frequency of social media use (Jalisco: AOR 1.21, 95% CI 0.99-1.47; and Southern California: AOR 1.41, 95% CI 1.15-1.97), although results for Jalisco were borderline statistically significant. Having best friends who used e-cigarettes (Jalisco: AOR 6.46, 95% CI 4.38-9.54; and Southern California: AOR 11.24, 95% CI 6.94-18.22) was associated with a higher likelihood of using e-cigarettes, and in Southern California only, females (AOR 0.50, 95% CI 0.34-0.72) had a lower likelihood of using e-cigarettes.

Models by each specific social media platform showed that only Instagram and TikTok were significantly associated with susceptibility in Jalisco, whereas TikTok was the only platform significantly associated with susceptibility in Southern California. In Jalisco, exposure to e-cigarette posts across all social media platforms was independently associated with past-month use, while for Southern California, exposure to e-cigarette posts on Instagram, TikTok, Snapchat, or Twitter was associated with susceptibility (Multimedia Appendix 1). In models pooling data from both countries, interactions between country and exposure to e-cigarette content were not significant for susceptibility (*χ*^2^_1_=1.13, *P*=.30) or e-cigarette use (*χ*^2^_1_= 0.26, *P*=.60; results not shown).

**Table 3:** Unadjusted and adjusted logistic models for past-month e-cigarette use, high school students in Jalisco and Southern California, 2022-2023.

Variables			Jalisco, Mexico (n=1418)									Southern California, United States (n=2953)						
			OR^a^ (95% CI)		*P* value		AOR^b^ (95% CI)		*P* value		OR (95% CI)			*P* value		AOR (95% CI)		*P* value
Frequency of using social media, index^c^			1.44 (1.20-1.72)		<.001		1.21 (0.99-1.47)		.05		2.00 (1.68-2.38)			<.001		1.41 (1.15-1.97)		.04
Frequency of e-cigarette exposure on social media, index^d^			1.62 (1.40-1.87)		<.001		1.49 (1.27-1.74)		<.001		1.94 (1.61-2.34)			<.001		1.51 (1.15-1.73)		.003
Age			1.09 (0.95-1.25)		.22		1.09 (0.94-1.27)		.31		1.24 (0.80-1.92)			.16		1.14 (0.79-1.64)		.45
**Sex**																		
	Male	*Ref* ^e^				*Ref*				*Ref*					*Ref*			
	Female	0.86 (0.65-1.15)		.32		1.03 (0.76-1.40)		.83		0.34 (0.25-0.47)			<.001		0.50 (0.34-0.72)		.002	
Family affluence scale, (0-9)			0.99 (0.94-1.07)		.97		1.04 (0.97-1.12)		.39		0.94 (0.85-1.03)			.18		0.94 (0.85-1.04)		.22
**Best friends’ use of e-cigarettes**																		
	None	*Ref*				*Ref*				*Ref*					*Ref*			
	1 or more	6.86 (4.67-10.07)		<.001		6.46 (4.38-9.54)		<.001		14.35 (9.01-22.87)			<.001		11.24 (6.94-18.22)		<.001	

^a^OR: odds ratio.

^b^AOR: adjusted odds ratio from model that includes all variables shown in table.

^c^Frequency of using social media represents the average of the frequency of using each social media platform, it ranges from (0-4).

^d^Frequency of seeing e-cigarette posts on social media index represents the average of the reported frequency of seeing e-cigarettes posts in each social media platform, ranges (0-4).

^e^*Ref*: reference value.

## Discussion

Aligned with our first hypothesis, we found high levels of self-reported use of and exposure to e-cigarette content through social media platforms in samples of high schoolers in Jalisco (Mexico) and Southern California (United States), particularly for YouTube, Instagram, and TikTok. However, these findings were surprising and contrary to our second hypothesis, given that Mexico bans commercial advertising for e-cigarettes across all channels, including social media platforms [32]. California has no prohibitions regarding online marketing; however, marketing and sales of all flavored tobacco products, except menthol, are prohibited, and starting in 2025, this ban is planned to be extended to online sales and include fines to ensure age verification [33,34]. In addition, e-cigarette products authorized by the FDA will not be able to market to the youth [35], although enforcement of marketing restrictions, particularly on social media platforms, remains challenging. We also found that past-month use of e-cigarettes was significantly lower in Southern California (139/2953, 4.7%) than in Jalisco (248/1418, 17.5%). Prevalence in Jalisco was substantially higher than in national surveys [36], although consistent with previous research in urban areas of Mexico [13], while the lower prevalence observed in Southern California aligns with data indicating lower e-cigarette use in California compared with other states [9]. More research is needed to understand the factors driving the difference in e-cigarette use prevalence between samples; better law enforcement, paired with media campaigns and prevention programs in schools, could play an important part in Southern California.

As illustrated in Figure 1, adolescents’ frequency of using specific social media platforms differed across the 2 samples, with more sizable differences for Facebook, which was higher in Jalisco. However, in both samples, the frequency of exposure to e-cigarette content through Facebook, as well as through Snapchat (only asked in Southern California) and WhatsApp (only asked in Jalisco), was low compared with TikTok, Instagram, and YouTube. A meta-analysis that examined associations between social media use and risky behaviors found that social media platforms that were introduced relatively early, like Facebook, had weaker associations than contemporary social media platforms when it comes to substance use [37]. In the past, Facebook has faced scrutiny for its impact on promoting unhealthy behaviors and targeting youth with advertisements, and in 2021, the platform limited the targeting options to 18-year-olds and older and banned all tobacco and nicotine products advertising [38]. That policy, along with Facebook use declining among the US youth [18] and other measures taken by California, could help explain the relatively low exposure reported in the Southern California sample. Additionally, the user guidelines across social media platforms prohibit any marketing and sales of any tobacco products in both countries; however, posts alluding to e-cigarettes and other substances can still be found. Studies are needed to characterize the content of e-cigarette–related posts across social media platforms, as such content may both promote and discourage e-cigarette use. Understanding the balance and nature of these messages is important not only for preventing dissemination of pro–e-cigarette content, but also for identifying content that may support prevention and cessation of e-cigarettes. For example, predominant topics in e-cigarette posts on Snapchat, one of the less popular social media platforms we studied, included messages about “health consequences” and “quitting” [39].

Higher exposure to e-cigarette posts was associated with higher use in both samples, as observed in previous studies. A meta-analysis that reviewed cross-sectional cohort studies and longitudinal studies found that those exposed to e-cigarette posts during the last 30-days are more likely to report current use [40]. Also consistent with other studies [41,42], we found that higher exposure to e-cigarette posts was associated with susceptibility to use e-cigarettes among students in both samples; however, the adjusted association remained statistically significant only for Jalisco. There are several possible explanations for the lack of association in Southern California. There was heterogeneity in effects across social media platforms (Multimedia Appendix 1); when examined individually, only TikTok had a significant association with susceptibility. Additionally, Southern California had a higher percentage of nonsusceptible students (Southern California: 2469/2953, 83.6% vs Jalisco: 830/1418, 58.5%), and possibly stronger social norms and/or higher levels of peer influence against e-cigarette use exist there. Indeed, fewer students reported having best friends who used e-cigarettes in Southern California compared with Mexico (785/2953, 24.7% vs 785/1418, 55.4%, respectively), which may have resulted in lower exposure to e-cigarette posts from their best friends that may have a greater impact on susceptibility than posts from people whom they do not know. In fact, high-school students in Southern California who had more friends who posted pictures of themselves partying or drinking alcohol online (via Facebook or MySpace [Viant Technology LLC]) were more likely to report that they smoked and used alcohol [43]. Although site-specific analyses suggested differences across sites, pooled analyses including a site-by-exposure interaction term did not indicate statistically significant effect modification by site (models not shown). In combination with minor measurement differences across sites, these results warrant cautious interpretation of between-site differences. Future research should try to understand whether sources of exposure to posts vary across different groups, cities, or countries, as this may help identify strategies to prevent such posts.

Although our data cannot ascertain the nature of the e-cigarette–related posts adolescents viewed on social media platforms, explicit and implicit promotion of e-cigarettes abounds. e-Cigarette marketing through social media platforms presents challenges for regulation. The World Health Organization recommends a comprehensive ban on Tobacco Advertising, Promotion, and Sponsorship, including efforts to limit digital content created in one country being broadcast in others [44-46]. However, for this to be effective, countries and platforms must be actively engaged and willing to collaborate. Differences in regulatory frameworks across and within countries pose a significant challenge. Additionally, only some content posted on social media platforms comes directly from the tobacco and vaping industry, and *influencer*- and user-generated content [47] will likely be harder to regulate, although requiring content moderation and age gates for specific types of content may help. Knowing that exposure to e-cigarette posts on social media platforms is associated with higher e-cigarette use among adolescents, countries should collaborate to protect their youth.

Peer influence is one of the most common drivers for substance use among adolescents, including for e-cigarettes [48-50]. Similar to other studies, having friends who use e-cigarettes was associated with a higher likelihood of susceptibility to and use of e-cigarettes in both samples, with stronger associations for students in Southern California. Longitudinal measures on (online) interactions among adolescents are much needed to better understand diffusion processes of tobacco products in adolescent peer networks [51]. The lack of association we found between family affluence and either susceptibility or use has been observed in other middle-income countries [52,53]. One possible explanation comes from diffusion of innovations theory [54], where, when a product is first introduced, those in higher socioeconomic status groups are more likely to know about and afford it; however, as the product becomes more popular, costs come down, and adoption extends across socioeconomic status groups. Indeed, as e-cigarettes became more popular, the global market transitioned from refillable devices to relatively cheaper pod systems to disposable devices, the last of which is now the most commonly used e-cigarette device among youth [55]. Additionally, in Mexico, these devices can be found on sale for $100 Mexican pesos (approximately US $5) on street markets or *tianguis*, which makes them affordable even to adolescents in lower socioeconomic status groups.

This first study to compare social media platforms use, exposures to e-cigarette content, and their associations with e-cigarette susceptibility and use among adolescents in Mexico and the United States has limitations. First, although measures used in each country were harmonized and have similar face validity, some measures were not identical across sites (eg, differences in question wording or response options; Multimedia Appendix 3). As a result, findings from cross-site comparisons should be interpreted with caution. Second, the temporal relationship between these social media platforms use, exposure to e-cigarette content, and susceptibility and use could not be determined. Selective attention to e-cigarette content is less likely to explain results around susceptibility than current use, as youth who have not initiated e-cigarette use might be less likely to actively seek content lowering their exposure. Additionally, our results align with previous cross-sectional and longitudinal studies, as well as with a large body of research showing tobacco marketing effects on youth [56]. Furthermore, our subsequent cognitive interviews with adolescents in Jalisco confirmed their confidence in distinguishing e-cigarette exposures by social media platforms. Third, our assessment of social media platforms was limited to those for which data were available on their popularity, with 2 social media platforms (WhatsApp in Southern California and Snapchat in Jalisco) not evaluated in both sites. Exclusion of social media platforms may have underestimated exposure and, potentially, attenuated observed associations. However, relative to the social media platforms assessed, WhatsApp and Snapchat are less popular in the sites where they were not assessed [18,57], suggesting that their omission is unlikely to have substantially altered the associations observed. Nevertheless, future studies may cast a broader net across social media platforms to more comprehensively characterize adolescents’ social media environments. Fourth, unmeasured confounders, such as mental health or other substance use, including among parents, were not included in the analyses due to a lack of harmonization. Finally, participation rates differed across study sites, in part due to differences in parental consent procedures. Active parental consent was required in Southern California, whereas passive (opt-out) consent was used in Jalisco, likely resulting in higher participation rates in the latter. The slightly lower participation rates in Southern California may have introduced differential selection bias based on parental engagement and potential confounders (eg, risk behaviors); however, we believe such biases would be minimal due to the relatively high participation rates in both sites. In addition, we surveyed students from convenience samples from public schools in both countries; therefore, the results may not generalize to broader populations of high schoolers, whether for the state or country. However, our findings are consistent with the prevalence of e-cigarette use found in previous studies that also involved self-administered questionnaires [13]. Additionally, these findings offer valuable preliminary insights into potential cross-country patterns of susceptibility and use of e-cigarettes across distinct regulatory and social contexts that warrant further investigation using population-representative data.

Exposure to social media and social media posts about e-cigarettes was high in both samples of high-school students and associated with susceptibility to and use of e-cigarettes. Despite Mexico having banned the sales and importation of e-cigarettes, susceptibility and current use were higher among adolescents in Jalisco than in Southern California, demonstrating that the ban alone is insufficient without proper enforcement. This cross-country comparison contributes to the literature highlighting how differences in regulatory environments and enforcement can influence youth exposure and behavior, reinforcing the need for coordinated, cross-border tobacco control strategies. Cross-national collaboration may be needed to limit adolescents’ exposure to pro–e-cigarette content and reduce use in both countries. Future research should prioritize population-based studies to monitor trends in youth exposure and use across diverse social media platforms and settings, while also evaluating how enforcement strategies, digital marketing regulations, and broader cultural and social contexts interact to shape adolescents’ susceptibility and use, to inform more effective public health policies.

## Appendix

Multimedia Appendix 1 Sensitivity analysis using logistic regression models for each social media platform.

Multimedia Appendix 2 Sensitivity analysis using multilevel logistic models with school as random effect.

Multimedia Appendix 3 Survey questions by study site.

## Abbreviations

AOR: adjusted odds ratio
ADVANCE: Assessing Developmental Patterns of Vaping, Alcohol, Nicotine, and Cannabis Use and Emotional Wellbeing
e-Cigarette: electronic cigarette
FAS: Family Affluence Scale
FDA: US Food and Drug Administration
REDCap: Research Electronic Data Capture

## Data Availability

Data will be made available at reasonable request to the corresponding author (JFT).

## References

[1] Freeman B, Watts C, Astuti PAS (2022). Global tobacco advertising, promotion and sponsorship regulation: What's old, what's new and where to next?. Tob Control.

[2] Miech RA, Johnson LD, Patrick ME, O'Malley PM, Bachman JG, Schulenberg JE (2023). Monitoring the Future National Survey Results on Drug Use, 1975-2022: Secondary School Students.

[3] Gentzke AS, Wang TW, Cornelius M, Park-Lee E, Ren C, Sawdey MD, Cullen KA, Loretan C, Jamal A, Homa DM (2022). Tobacco Product Use and Associated Factors Among Middle and High School Students - National Youth Tobacco Survey, United States, 2021. MMWR Surveill Summ.

[4] Dewhirst T (2021). Co-optation of harm reduction by Big Tobacco. Tob Control.

[5] (2019). Tobacco 21 | FDA. U.S. Food and Drug Administration.

[6] How FDA regulates vapes. Food and Drug Administration.

[7] Birdsey J, Cornelius M, Jamal A, Park-Lee E, Cooper MR, Wang J, Sawdey MD, Cullen KA, Neff L (2023). Tobacco Product Use Among U.S. Middle and High School Students - National Youth Tobacco Survey, 2023. MMWR Morb Mortal Wkly Rep.

[8] Gentzke AS, Creamer M, Cullen KA, Ambrose BK, Willis G, Jamal A, King BA (2019). Vital Signs: Tobacco Product Use Among Middle and High School Students - United States, 2011-2018. MMWR Morb Mortal Wkly Rep.

[9] Tobacco use in California 2023. Truth Initiative.

[10] Cámara de Diputados del Congreso de la Nación [Chamber of Deputies National Congress] (2008). Ley general para el control del tabaco [General Tobacco Control Law]. Cámara de Diputados del Congreso de la Nación.

[11] Cámara de diputados del Congreso de la Nación [Chamber of Deputies National Congress] (2008). Decreto por el que se expide la ley general para el control del tabaco; y deroga y reforma diversas disposiciones de la Ley general de salud. Diario Oficial de la Federación.

[12] Zavala-Arciniega, Luis (2023). Use of tobacco and new products during the COVID-19 pandemic in Mexico: A social determinants perspective.

[13] Barrientos-Gutierrez I, Lozano P, Arillo-Santillan E, Morello P, Mejia R, Thrasher JF (2019). "Technophilia": A new risk factor for electronic cigarette use among early adolescents?. Addict Behav.

[14] Vidaña-Pérez D, Gallegos-Carrillo K, Barrientos-Gutierrez I, Cruz-Jiménez L, Rodríguez-Bolaños R, Arillo-Santillán E, Thrasher JF (2024). Prevalence and correlates of e-cigarette source and use of e-cigarettes with nicotine: A case study of Mexico, where e-cigarettes are banned. Int J Drug Policy.

[15] Zavala-Arciniega L, Reynales-Shigematsu LM, Lozano P, Rodríguez-Andrade MA, Arillo-Santillán E, Thrasher JF (2018). Patterns of awareness and use of electronic cigarettes in Mexico, a middle-income country that bans them: Results from a 2016 national survey. Prev Med.

[16] (2024). Digital 2024: Global overview report. We are Social, Meltwater.

[17] Instituto Nacional de Estadística y Geografia (INEGI) (2021). Encuesta Nacional sobre Disponibilidad y Uso de Tecnologías de la Información en los Hogares (ENDUTIH). National Survey on Availability and Use of Information Technologies in Households Press Release. Mexico City. Instituto Nacional de Estadística y Geografia (INEGI); Instituto Federal de Telecomunicaciones.

[18] (2022). Teens, social media and technology. Pew Research Center.

[19] (2024). Centers for Disease Control and Prevention.

[20] Cho YJ, Thrasher JF, Driezen P, Hitchman SC, Reid JL, Hammond D (2022). Trends in exposure to and perceptions of e-cigarette marketing among youth in England, Canada and the United States between 2017 and 2019. Health Educ Res.

[21] (2023). Results from the annual national youth tobacco survey. Food and Drug Administration (FDA).

[22] Lee J, Suttiratana SC, Sen I, Kong G (2023). E-Cigarette Marketing on Social Media: A Scoping Review. Curr Addict Rep.

[23] Zheng X, Li W, Li R, Yang M, Lin HC (2024). Exposure to user-generated e-cigarette content on social media associated with greater vulnerability to e-cigarette use among youth non-users. Addict Behav.

[24] Duke JC, Allen JA, Eggers ME, Nonnemaker J, Farrelly MC (2016). Exploring Differences in Youth Perceptions of the Effectiveness of Electronic Cigarette Television Advertisements. Nicotine Tob Res.

[25] Mantey DS, Cooper MR, Clendennen SL, Pasch KE, Perry CL (2016). E-Cigarette Marketing Exposure Is Associated With E-Cigarette Use Among US Youth. J Adolesc Health.

[26] Vassey J, Valente T, Barker J, Stanton C, Li D, Laestadius L, Cruz TB, Unger JB (2023). E-cigarette brands and social media influencers on Instagram: a social network analysis. Tob Control.

[27] Leventhal AM, Strong DR, Kirkpatrick MG, Unger JB, Sussman S, Riggs NR, Stone MD, Khoddam R, Samet JM, Audrain-McGovern J (2015). Association of electronic cigarette use with initiation of combustible tobacco product smoking in early adolescence. JAMA.

[28] Pierce JP, Farkas AJ, Evans N, Gilpin E (1995). An improved surveillance measure for adolescent smoking?. Tob Control.

[29] Kehoe S, O'Hare L (2010). The reliability and validity of the family affluence scale. Effective Education.

[30] Torsheim T, Currie C, Boyce W, Kalnins I, Overpeck M, Haugland S (2004). Material deprivation and self-rated health: A multilevel study of adolescents from 22 European and North American countries. Soc Sci Med.

[31] Pérez A, Thrasher J, Monzón JC, Arillo-Santillán E, Barnoya J, Mejía R (2021). La escala de afluencia familiar en la investigación sobre inequidades sociales en salud en adolescentes latinoamericanos. Salud Publica Mex.

[32] (2023). Campaign for Tobacco Free Kids.

[33] California Legislative Information (2021). California Code, BPC 22963. California Code.

[34] Game over: Ending internet sales of commercial tobacco. Public Health Law Center.

[35] Youth tobacco prevention plan. Food and Drug Administration (FDA).

[36] Shamah-Levy T, Romero-Martinez M, Barrientos-Gutierrez T, Cuevas-Nasu L, Bautista-Arredondo S, Colchero MA, Gaona-Pineda EB, Lazcano-Ponce E, Martinez-Barnetche J, Alpuche-Arana C, Rivera-Dommarco J (2022). Encuesta Nacional de Salud y Nutrición 2021 sobre COVID-19. Resultados nacionales. Instituto Nacional de Salud Publica.

[37] Vannucci A, Simpson EG, Gagnon S, Ohannessian CM (2020). Social media use and risky behaviors in adolescents: A meta-analysis. J Adolesc.

[38] (2021). Facebook is changing how advertisers can reach young people. Meta for Business.

[39] Majmundar A, Chu M, Perez C, Hoang Y, Yuan J, Unger JB, Allem J (2022). Tobacco and cannabis use advertisements targeting adolescents and young adults on Snapchat in 2019. Prev Med Rep.

[40] Rutherford BN, Lim CCW, Cheng B, Sun T, Vu GT, Johnson B, Chung J, Huang S, Leung J, Stjepanović D, Connor JP, Chan GCK, Daniel Paul Ashley (2023). Viral Vaping: A systematic review and meta analysis of e-cigarette and Tobacco-Related social media content and its influence on youth behaviours and attitudes. Addict Behav.

[41] Lee J, Krishnan-Sarin S, Kong G (2023). Social Media Use and Subsequent E-Cigarette Susceptibility, Initiation, and Continued Use Among US Adolescents. Prev Chronic Dis.

[42] Pérez A, Spells CE, Bluestein MA, Harrell MB, Hébert ET (2022). The longitudinal impact of seeing and posting tobacco-related social media on tobacco use behaviors among Youth (Aged 12-17): Findings from the 2014-2016 population assessment of tobacco and health (PATH) study. Tob Use Insights.

[43] Huang GC, Unger JB, Soto D, Fujimoto K, Pentz MA, Jordan-Marsh M, Valente TW (2014). Peer influences: The impact of online and offline friendship networks on adolescent smoking and alcohol use. J Adolesc Health.

[44] What is cross-border TAPS? | WHO FCTC. World Health Organization.

[45] (2024). Guidelines for implementation article 13 and specific guidelines to address crossborder tobacco advertising, promotion and sponsorship and the depiction of tobacco in entertainment media for implementation of article 13 of the WHO FCTC. World Health Organization.

[46] (2020). Tobacco: Industry tactics to attract younger generations 2020. World Health Organization.

[47] Smith MJ, Buckton C, Patterson C, Hilton S (2023). User-generated content and influencer marketing involving e-cigarettes on social media: A scoping review and content analysis of youTube and instagram. BMC Public Health.

[48] Groom AL, Vu TT, Landry RL, Kesh A, Hart JL, Walker KL, Wood LA, Robertson RM, Payne TJ (2021). The influence of friends on teen vaping: A mixed-methods approach. Int J Environ Res Public Health.

[49] Henneberger Ak, Mushonga DR, Preston AM (2020). Peer influence and adolescent substance use: A systematic review of dynamic social network research. Adolescent Res Rev.

[50] Valente TW, Piombo SE, Edwards KM, Waterman EA, Banyard VL (2023). Social network influences on adolescent E-cigarette use. Subst Use Misuse.

[51] Kitts JA, Leal DF (2021). What is(n't) a friend? Dimensions of the friendship concept among adolescents. Soc Networks.

[52] Vidaña-Pérez D, Mus S, Monzón J, Dávila G, Fahsen N, Barnoya J, Thrasher JF (2024). Factors associated with the changes in smoking and electronic cigarette use in adolescents during the COVID-19 pandemic: A longitudinal analysis. J Adolesc Health.

[53] Lozano P, Arillo-Santillán E, Barrientos-Gutíerrez I, Reynales Shigematsu LM, Thrasher JF (2019). E-cigarette social norms and risk perceptions among susceptible adolescents in a country that bans E-cigarettes. Health Educ Behav.

[54] Valente TW, Carrington PJ, Sctt J, Wasserman S (2005). Network models and methods for studying the diffusion of innovations. Models and Methods in Social Network Analysis.

[55] Hammond D, Reid JL, Burkhalter R, East K (2025). Use of disposable e-cigarettes among youth who vape in Canada, England and the United States: Repeat cross-sectional surveys, 2017-2023. Addiction.

[56] (2008). The role of the media in promoting and reducing tobacco use. National Cancer Institute.

[57] (2023). Digital 2022: Global overview report. We are Social, Hootsuite.

