# Supplementary material for: Exposure to e-Cigarette Posts Across Social Media Platforms and Its Associations With Susceptibility and e-Cigarette Use: Comparative Cross-Sectional Study of High Schoolers in Jalisco (Mexico) and Southern California (United States)
Source: JMIR Pediatr Parent. 2026 Mar 13;9:e85376. doi: 10.2196/85376 (PMC13032094; doi:10.2196/85376)
Supplement: Multimedia Appendix 1 [file pediatrics_v9i1e85376_app1.docx]

Multimedia Appendix1:

The following table shows the adjusted logistic models for susceptibility to use electronic cigarettes and the self-reported frequency of exposure to electronic cigarettes posts on social media adjusted by covariates. Each column represents a separate model. (Jalisco)

| **Table 1A. Separate Adjusted logistic models for the susceptibility to use electronic cigarettes and the self-reported exposure to e-cigarette posts on each social media platform.** | | | | | | | | | | | | | | |
| --- | --- | --- | --- | --- | --- | --- | --- | --- | --- | --- | --- | --- | --- | --- |
|  | **Facebook** | | **Instagram** | | **Youtube** | | **TikTok** | | **WhatsApp** | | **Twitter** | | **Twitch** | |
|  | **AOR** | **(IC 95%)** | **AOR** | **(IC 95%)** | **AOR** | **(IC 95%)** | **AOR** | **(IC 95%)** | **AOR** | **(IC 95%)** | **AOR** | **(IC 95%)** | **AOR** | **(IC 95%)** |
| **E-cigarette posts index ^ꝉ^** | 1.09 | (0.96 - 1.22) | **1.14** | **(1.01 - 1.27)** | 1.13 | (0.99 - 1.29) | **1.14** | **(1.03 - 1.27)** | 1.05 | (0.93 - 1.18) | 1.15 | (0.96 - 1.38) | 1.15 | (0.96 - 1.38) |
| **Social media use index^±^** | 1.15 | (0.96 - 1.38) | 1.14 | (0.95 - 1.36) | 1.15 | (0.96 - 1.38) | 1.13 | (0.94 - 1.35) | 1.17 | (0.97 - 1.39) | 1.15 | (0.96 - 1.38) | 1.14 | (0.95 - 1.37) |
| **Age** | **0.62** | **(0.47 - 0.83)** | **0.63** | **(0.47 - 0.84)** | **0.62** | **(0.46 - 0.82)** | **0.64** | **(0.48 - 0.85)** | **0.62** | **(0.47 - 0.83)** | **0.61** | **(0.46 - 0.81)** | **0.61** | **(0.46 - 0.81)** |
| **Sex** |  |  |  |  |  |  |  |  |  |  |  |  |  |  |
| Female | Ref. | | Ref. | | Ref. | | Ref. | | Ref. | | Ref. | | Ref. | |
| Male | 1.05 | (0.92 - 1.21) | 1.05 | (0.92 - 1.21) | 1.06 | (0.92 - 1.22) | 1.06 | (0.93 - 1.22) | 1.06 | (0.92 - 1.21) | 1.05 | (0.92 - 1.21) | 1.05 | (0.92 - 1.21) |
| **Family affluence scale** | 0.98 | (0.92 - 1.05) | 0.98 | (0.92 - 1.05) | 0.99 | (0.92 - 1.05) | 0.98 | (0.92 - 1.05) | 0.99 | (0.92 - 1.05) | 0.98 | (0.92 - 1.05) | 0.98 | (0.92 - 1.05) |
| **Friends use ecigs** |  |  |  |  |  |  |  |  |  |  |  |  |  |  |
| None | Ref. | | Ref. | | Ref. | | Ref. | | Ref. | | Ref. | | Ref. | |
| 1 or more | **3.83** | **(2.89 - 5.06)** | **3.78** | **(2.86 - 5.00)** | **3.84** | **(2.91 - 5.08)** | **3.84** | **(2.91 - 5.08)** | **3.84** | **(2.91 - 5.08)** | **3.84** | **(2.90 - 5.07)** | **3.86** | **(2.92 - 5.11)** |

**^ꝉ^** Frequency of using social media index represents the average of the reported frequency of using each social media platform, it ranges from (0-4)

**^±^** Frequency of seeing e-cigarette posts on social media index represents the average of the reported frequency of seeing e-cigarettes posts in each social media platform, it ranges from (0-4).

The following table shows the adjusted logistic models for current electronic cigarette use and the self-reported frequency of exposure to electronic cigarettes posts on social media adjusted by covariates. Each column represents a separate model. (Jalisco)

| **Table 2A. Separate Adjusted logistic models for current electronic cigarette use and self-reported exposure to e-cigarette posts on each social media platform.** | | | | | | | | | | | | | | | | |
| --- | --- | --- | --- | --- | --- | --- | --- | --- | --- | --- | --- | --- | --- | --- | --- | --- |
|  | **Facebook** | | **Instagram** | | **Youtube** | | **TikTok** | | **WhatsApp** | | **Twitter** | | | **Twitch** | | |
|  | **AOR** | **(IC 95%)** | **AOR** | **(IC 95%)** | **AOR** | **(IC 95%)** | **AOR** | **(IC 95%)** | **AOR** | **(IC 95%)** | **AOR** | **(IC 95%)** | **AOR** | | **(IC 95%)** |  |
| **E-cigarette posts index ^ꝉ^** | **1.23** | **(1.10 - 1.38)** | **1.27** | **(1.14 - 1.42)** | **1.20** | **(1.06 - 1.36)** | **1.30** | **(1.17 - 1.44)** | **1.27** | **(1.13 - 1.42)** | **1.34** | **(1.15 - 1.56)** | **1.25** | | **(1.07 - 1.46)** |  |
| **Social media use index^±^** | **1.26** | **(1.04 - 1.53)** | **1.24** | **(1.02 - 1.50)** | **1.28** | **(1.06 - 1.55)** | **1.23** | **(1.01 - 1.49)** | **1.28** | **(1.06 - 1.55)** | **1.25** | **(1.03 - 1.51)** | **1.26** | | **(1.04 - 1.52)** |  |
| **Age** | 1.05 | (0.78 - 1.43) | 1.06 | (0.78 - 1.44) | 1.01 | (0.74 - 1.37) | 1.10 | (0.95 - 1.28) | 1.07 | (0.78 - 1.45) | 0.97 | (0.71 - 1.32) | 0.99 | | (0.73 - 1.35) |  |
| **Sex** |  |  |  |  |  |  |  |  |  |  |  |  |  | |  |  |
| Female | Ref. | | Ref. | | Ref. | | Ref. | | Ref. | | Ref. | | | Ref. | | |
| Male | 1.08 | (0.93 - 1.25) | 1.09 | (0.94 - 1.26) | 1.09 | (0.94 - 1.26) | 1.10 | (0.95 - 1.28) | 1.09 | (0.94 - 1.26) | 1.08 | (0.93 - 1.25) | 1.08 | | (0.93 - 1.25) |  |
| **Family affluence scale** | 1.04 | (0.97 - 1.11) | 1.04 | (0.97 - 1.12) | 1.04 | (0.97 - 1.12) | 1.04 | (0.97 - 1.12) | 1.04 | (0.97 - 1.12) | 1.04 | (0.97 - 1.12) | 1.04 | | (0.97 - 1.11) |  |
| **Friends use ecigs** |  |  |  |  |  |  |  |  |  |  |  |  |  | |  |  |
| None | Ref. | | Ref. | | Ref. | | Ref. | | Ref. | | Ref. | | | Ref. | | |
| 1 or more | **6.52** | **(4.42 - 9.62)** | **6.38** | **(4.32 - 9.41)** | **6.62** | **(4.49 - 9.76)** | **6.58** | **(4.46 - 9.72)** | **6.50** | **(4.41 - 9.59)** | **6.55** | **(4.44 - 9.66)** | **6.67** | | **(4.52 - 9.83)** |  |

**^ꝉ^** Frequency of using social media index represents the average of the reported frequency of using each social media platform, it ranges from (0-4)

**^±^** Frequency of seeing e-cigarette posts on social media index represents the average of the reported frequency of seeing e-cigarettes posts in each social media platform, it ranges from (0-4).

The following table shows the adjusted logistic models for electronic cigarette susceptibility use and the self-reported frequency of exposure to electronic cigarettes posts on social media adjusted by covariates. Each column represents a separate model. (SoCal)

| **Table 3A. Separate adjusted logistic models for the susceptibility to use electronic cigarettes and self-reported exposure to e-cigarette posts on each social media platform.** | | | | | | | | | | | | | | | |
| --- | --- | --- | --- | --- | --- | --- | --- | --- | --- | --- | --- | --- | --- | --- | --- |
|  | **Facebook** | | **Instagram** | | **Youtube** | | **TikTok** | | **Snapchat** | | **Twitter** | | **Twitch** | | |
|  | **AOR** | **(IC 95%)** | **AOR** | **(IC 95%)** | **AOR** | **(IC 95%)** | **AOR** | **(IC 95%)** | **AOR** | **(IC 95%)** | **AOR** | **(IC 95%)** | **AOR** | **(IC 95%)** |  |
| **E-cigarette posts index ^ꝉ^** | 0.67 | (0.44 - 1.03) | 1.08 | (0.97 - 1.20) | 1.01 | (0.89 - 1.14) | **1.17** | **(1.05 - 1.29)** | 1.01 | (0.87 - 1.15) | 1.17 | (0.99 - 1.38) | 0.89 | (0.61 - 1.29) |  |
| **Social media use index^±^** | **1.79** | **(1.45 - 2.26)** | **1.88** | **(1.50 - 2.36)** | **1.9** | **(1.52 - 2.39)** | **1.78** | **(1.42 - 2.23)** | **1.89** | **(1.51 - 2.37)** | **1.17** | **(1.38 - 2.21)** | **1.92** | **(1.53 - 2.41)** |  |
| **Age** | 0.99 | (0.77 - 1.28) | 1.01 | (0.80 - 1.29) | 1.07 | (0.83 - 1.37) | 1.03 | (0.81 - 1.30) | 0.98 | (0.77 - 1.24) | 1.06 | (0.83 - 1.36) | 0.97 | (0.76 - 1.23) |  |
| **Sex** |  |  |  |  |  |  |  |  |  |  |  |  |  |  |  |
| Male | Ref. | | Ref. | | Ref. | | Ref. | | Ref. | | Ref. | | Ref. | | |
| Female | **0.65** | **(0.48 - 0.87)** | **0.55** | **(0.41 - 0.73)** | **0.52** | **(0.39 - 0.69)** | **0.61** | **(0.46 - 0.82)** | **0.62** | **(0.46 - 0.82)** | **0.64** | **(0.48 - 0.86)** | **0.57** | **(0.42 - 0.77)** |  |
| **Family affluence scale** | 1.01 | (0.93 - 1.09) | 1.03 | (0.96 - 1.12) | 1.01 | (0.93 - 1.09) | 1.07 | (0.99 - 1.16) | 1.06 | (0.97 - 1.14) | 1.04 | (0.96 - 1.12) | 1.05 | (0.96 - 1.13) |  |
| **Friends use ecigs** |  |  |  |  |  |  |  |  |  |  |  |  |  |  |  |
| None | Ref. | | Ref. | | Ref. | | Ref. | | Ref. | | Ref. | | Ref. | | |
| 1 or more | **3.57** | **(2.68 - 4.75)** | **3.13** | **(2.37 - 4.12)** | **3.35** | **(2.51 - 4.48)** | **3.29** | **(2.51 - 4.34)** | **3.4** | **(2.56 - 4.52)** | **3.24** | **(2.42 - 4.34)** | **3.19** | **(2.42 - 4.20)** |  |

**^ꝉ^** Frequency of using social media index represents the average of the reported frequency of using each social media platform, it ranges from (0-4).

**^±^** Frequency of seeing e-cigarette posts on social media index represents the average of the reported frequency of seeing e-cigarettes posts in each social media platform, it ranges from (0-4).

The following table shows the adjusted logistic models for current electronic cigarette use and the self-reported frequency of exposure to electronic cigarettes posts on social media adjusted by covariates. Each column represents a separate model. (SoCal)

| **Table 4A. Separate adjusted logistic models for the current use electronic cigarettes and self-reported exposure to e-cigarette posts on each social media platform**. | | | | | | | | | | | | | | | | |
| --- | --- | --- | --- | --- | --- | --- | --- | --- | --- | --- | --- | --- | --- | --- | --- | --- |
|  | **Facebook** | | **Instagram** | | **Youtube** | | **TikTok** | | **Snapchat** | | **Twitter** | | **Twitch** | | |  |
|  | **AOR** | **(IC 95%)** | **AOR** | **(IC 95%)** | **AOR** | **(IC 95%)** | **AOR** | **(IC 95%)** | **AOR** | **(IC 95%)** | **AOR** | **(IC 95%)** | **AOR** | **(IC 95%)** |  |  |
| **E-cigarette posts index ^ꝉ^** | 1.18 | (0.83 - 1.68) | **1.37** | **(1.17 - 1.59)** | 1.07 | (0.88 - 1.28) | **1.24** | **(1.07 - 1.45)** | **1.30** | **(1.10 - 1.54)** | **1.27** | **(1.01 - 1.60)** | 1.33 | (0.81 - 2.21) |  |  |
| **Social media use index^±^** | **1.51** | **(1.05 - 2.14)** | **1.51** | **(1.06 - 2.16)** | **1.48** | **(1.02 - 2.15)** | 1.27 | (0.90 - 1.81) | **1.47** | **(1.00 - 2.14)** | 1.36 | (0.94 - 1.97) | 1.38 | (0.98 - 1.95) |  |  |
| **Age** | 1.12 | (0.76 - 1.67) | 1.3 | (0.88 - 1.92) | 1.41 | (0.92 - 2.17) | 1.17 | (0.81 - 1.72) | 1.13 | (0.75 - 1.70) | 1.05 | (0.71 - 1.54) | 1.08 | (0.74 - 1.57) |  |  |
| **Sex** |  |  |  |  |  |  |  |  |  |  |  |  |  |  |  |  |
| Male | Ref. | | Ref. | | Ref. | | Ref. | | Ref. | | Ref. | | Ref. | | |  |
| Female | **0.53** | **(0.32 - 0.86)** | **0.53** | **(0.33 - 0.86)** | **0.48** | **(0.28 - 0.80)** | **0.48** | **(0.29 - 0.78)** | **0.54** | **(0.32 - 0.90)** | **0.43** | **(0.25 - 0.73)** | **0.59** | **(0.36 - 0.97)** |  |  |
| **Family affluence scale** | 0.95 | (0.85 - 1.07) | 0.99 | (0.89 - 1.12) | 1.01 | (0.89 - 1.15) | 0.96 | (0.86 - 1.07) | 0.96 | (0.85 - 1.09) | 1.03 | (0.91 - 1.16) | 0.94 | (0.84 - 1.04) |  |  |
| **Friends use ecigs** |  |  |  |  |  |  |  |  |  |  |  |  |  |  |  |  |
| None | Ref. | | Ref. | | Ref. | | Ref. | | Ref. | | Ref. | | Ref. | | |  |
| 1 or more | **14.04** | **(8.29 - 23.76)** | **10.7** | **(6.50 - 17.62)** | **12.66** | **(7.29 - 21.97)** | **11.69** | **(7.01 - 19.23)** | **13.43** | **(7.67 - 23.49)** | **11.98** | **(7.12 - 20.16)** | **14.95** | **(8.81 - 25.39)** |  |  |

**^ꝉ^** Frequency of using social media index represents the average of the reported frequency of using each social media platform, it ranges from (0-4).

**^±^** Frequency of seeing e-cigarette posts on social media index represents the average of the reported frequency of seeing e-cigarettes posts in each social media platform, it ranges from (0-4).
