# Supplementary material for: Exposure to e-Cigarette Posts Across Social Media Platforms and Its Associations With Susceptibility and e-Cigarette Use: Comparative Cross-Sectional Study of High Schoolers in Jalisco (Mexico) and Southern California (United States)
Source: JMIR Pediatr Parent. 2026 Mar 13;9:e85376. doi: 10.2196/85376 (PMC13032094; doi:10.2196/85376)
Supplement: Multimedia Appendix 2 [file pediatrics_v9i1e85376_app2.docx]

| Appendix 2. Multilevel models clustered by school.  **Supplementary table 1. Adjusted multilevel models for susceptibility to use e-cigarettes clustered by school.** | | | | | | | | | | | | | | | |
| --- | --- | --- | --- | --- | --- | --- | --- | --- | --- | --- | --- | --- | --- | --- | --- |
|  | **Jalisco, Mexico (n=1,170)** | | | | | | |  | **Southern California (n=2,695)** | | | | |  |  |
|  | **Odds ratio** | **95% CI** | | | |  | **P-value** |  | **Odds ratio** | **95% CI** | | | |  | **P-value** |
| **Frequency of using social media, index** | 1.13 | ( | 0.94 | , | 1.36 | ) | .181 |  | 1.85 | ( | 1.49 | , | 2.28 | ) | <.001 |
| **Frequency of e-cigarette exposure on social media, index** | 1.21 | ( | 1.02 | , | 1.44 | ) | .027 |  | 1.15 | ( | 0.94 | , | 1.40 | ) | .185 |
| **Age** | 1.05 | ( | 0.91 | , | 1.21 | ) | .483 |  | 1.02 | ( | 0.81 | , | 1.28 | ) | .849 |
| **Sex** |  |  |  |  |  |  |  |  |  |  |  |  |  |  |  |
| Male |  |  |  |  |  |  |  |  |  |  |  |  |  |  |  |
| Female | 0.62 | ( | 0.47 | , | 0.83 | ) | <.001 |  | 0.60 | ( | 0.47 | , | 0.78 | ) | <.001 |
| **Family affluence scale** | 0.99 | ( | 0.92 | , | 1.05 | ) | .665 |  | 1.06 | ( | 0.99 | , | 1.14 | ) | .104 |
| **Friends use ecigs** |  |  |  |  |  |  |  |  |  |  |  |  |  |  |  |
| None |  |  |  |  |  |  |  |  |  |  |  |  |  |  |  |
| 1 or more | 3.86 | ( | 2.91 | , | 5.13 | ) | <.001 |  | 3.33 | ( | 2.59 | , | 4.28 | ) | <.001 |

| **Supplementary table 2. Adjusted multilevel models for current e-cigarette use clustered by school.** | | | | | | | | | | | | | | | | | | |
| --- | --- | --- | --- | --- | --- | --- | --- | --- | --- | --- | --- | --- | --- | --- | --- | --- | --- | --- |
|  | **Jalisco, Mexico (n=1,418)** | | | | | | |  | **Southern California (n=2,953)** | | | | | | | | | |
|  | **Odds ratio** | **95% CI** | | | |  | **P-value** |  | **Odds ratio** | **95% CI** | | | | |  | | **P-value** | |
| **Frequency of using social media, index** | 1.22 | ( | 1.00 | , | 1.48 | ) | .049 |  | 1.44 | ( | 1.03 | , | 2.00 | ) | | .032 | |  |
| **Frequency of e-cigarette exposure on social media, index** | 1.47 | ( | 1.25 | , | 1.73 | ) | <.001 |  | 1.48 | ( | 1.12 | , | 1.96 | ) | | .005 | |  |
| **Age** | 1.09 | ( | 0.93 | , | 1.28 | ) | .271 |  | 1.13 | ( | 0.76 | , | 1.68 | ) | | .550 | |  |
| **Sex** |  |  |  |  |  |  |  |  |  |  |  |  |  |  | |  | |  |
| Male |  |  |  |  |  |  |  |  |  |  |  |  |  |  | |  | |  |
| Female | 1.05 | ( | 0.76 | , | 1.44 | ) | .759 |  | 0.49 | ( | 0.31 | , | 0.76 | ) | | <.001 | |  |
| **Family affluence scale** | 1.04 | ( | 0.97 | , | 1.12 | ) | .300 |  | 0.94 | ( | 0.85 | , | 1.04 | ) | | .211 | |  |
| **Friends use ecigs** |  |  |  |  |  |  |  |  |  |  |  |  |  |  | |  | |  |
| None |  |  |  |  |  |  |  |  |  |  |  |  |  |  | |  | |  |
| 1 or more | 6.22 | ( | 4.17 | , | 9.28 | ) | <.001 |  | 10.40 | ( | 6.61 | , | 16.36 | ) | | <.001 | |  |
