# Supplementary material for: Exposure to e-Cigarette Posts Across Social Media Platforms and Its Associations With Susceptibility and e-Cigarette Use: Comparative Cross-Sectional Study of High Schoolers in Jalisco (Mexico) and Southern California (United States)
Source: JMIR Pediatr Parent. 2026 Mar 13;9:e85376. doi: 10.2196/85376 (PMC13032094; doi:10.2196/85376)
Supplement: Multimedia Appendix 3 [file pediatrics_v9i1e85376_app3.docx]

Appendix 3. List of each survey questions with their original wording.

| **ESMAV-Jalisco** | **ADVANCE- SoCal** |
| --- | --- |
| How often do you visit the following social media sites?  (¿*Qué tan seguido usas las siguientes redes sociales?)*  0=“I don’t use this social media”, 1=“once a month or less”, 2=“weekly”, 3=“daily”, 4=“several times a day”, 5=“I don’t know”. | How often do you visit the following social media sites?  0=“I don’t use this social media”, 1=“once a month or less”, 2=“weekly”, 3=“daily”, 4=“several times a day”, 5=“I don’t know”. |
| Thinking about the social media platforms that you use, how often do you see posts, stories, or status about e-cigarettes or vapes (a device with a battery and a coil that heats a liquid , vaporizing it, which may contain nicotine or other substances)?  (Pensando en las redes sociales que utilizas, ¿Qué tan seguido ves posts, publicaciones, historias o estados que hagan referencia a  cigarros electrónicos o vapeadores (un aparato con una pila y una resistencia que calienta un líquido, vaporizándolo, que puede contener nicotina u otras sustancias)?  (Responses: 1=“I don’t use this social, 2=”never”, 3=“once a month or less”, 4=“weekly”, 5=“daily”, 6=“several times a day”. | How often do you see posts about e-cigarettes and nicotine vaping products on the following social media sites?  (Responses: 0=“never”, 1= “monthly or less”, 2=”weekly”, 3= “daily”, to 4=“several times a day” and 5=“ I don’t know” |
| In the last 30 days, how many total days have you used any electronic cigarettes?  ***En los últimos 30 días,****¿Cuántos días usaste cigarros electrónicos?* | In the last 30 days, how many total days have you used any electronic cigarette with nicotine? |
| Do you think that at some point during the next 12 months, you will use an e-cigarette? *(¿Crees que, en algún momento durante los próximos 12 meses, usarás un cigarro electrónico?)*  (0=“definitely not; 1="probably not,” “probably yes,” or “definitely yes”). | Do you think you would electronic cigarettes for vaping nicotine in the next year…?  (0=“definitely not; 1="probably not,” “probably yes,” or “definitely yes”). |
| The FAS includes the following questions:  1) How many cars or trucks does your family own (0, 1, 2, 3 or more) 2) Do you have a room for yourself? (0, 1) 3) During the last 12 months, how many times did you go on vacation with your family? (0, 1, 2, 3 or more) 4) How many computers does your family have? (0, 1, 2, 3 or more) | The FAS includes the following questions:   1. Does your family own a car, van or truck? (0, 1, 2, 3 or more) 2. Do you have your own bedroom for yourself? (0, 1) 3. During the past 12 months, how many times did you travel away on holiday with your family? (0, 1, 2, 3 or more) 4. How many computers does your family own? (0, 1, 2, 3 or more) |
| “Of your five best friends, how many use electronic cigarettes?” (De tus cinco mejores amigos (as) ¿Cuántos de ellos o ellas usan cigarro electrónico?) | “How many of your five (5) closest friends use electronic cigarettes for vaping nicotine?” |
| How old are you? (options: <12, 13, 14, 15, 16, 17, 18, 19, and >19) | How old are you? (Continuous) |
| Sex (Male, female) | What was your sex assigned at birth? (Male, female) |
